# Supplementary material for: Efficient Synthesis of High-Performance Anion Exchange Membranes by Applying Clickable Tetrakis(dialkylamino)phosphonium Cations
Source: Polymers (Basel). 2023 Jan 9;15(2):352. doi: 10.3390/polym15020352 (PMC9862753; doi:10.3390/polym15020352)
Supplement: Supplementary file 1 [file polymers-15-00352-s001.zip › polymers-2058091-supplementary.pdf]

Supporting Information: An efficient synthesis of high-performance anion exchange membranes by applying clickable tetrakis(dialkylamino)phosphonium cations

Characterization results

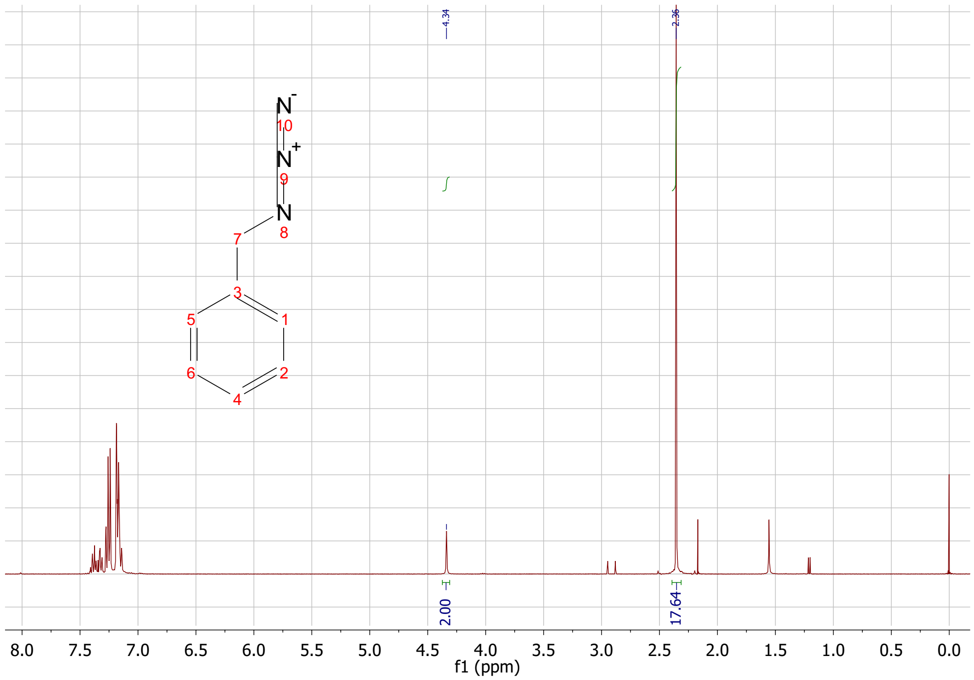

Figure S1: ^{1}H NMR (400 MHz, $CDCl_3$) spectra of benzyl azide solution in toluene.

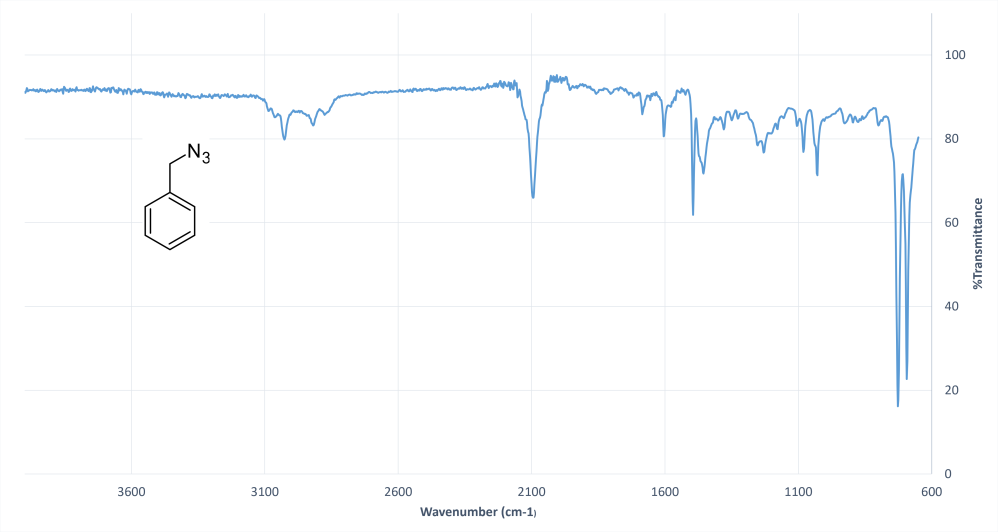

Figure S2: FTIR spectra of benzyl azide solution in toluene.

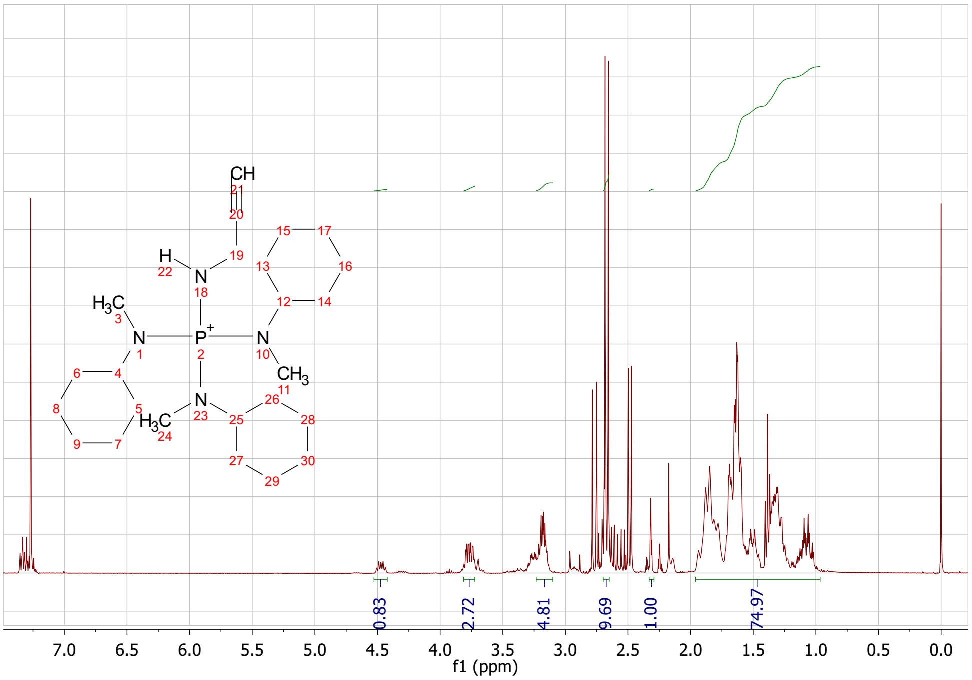

Figure S3: ^{1}H NMR (400 MHz, $CDCl_3$) spectra of compound **1'**.

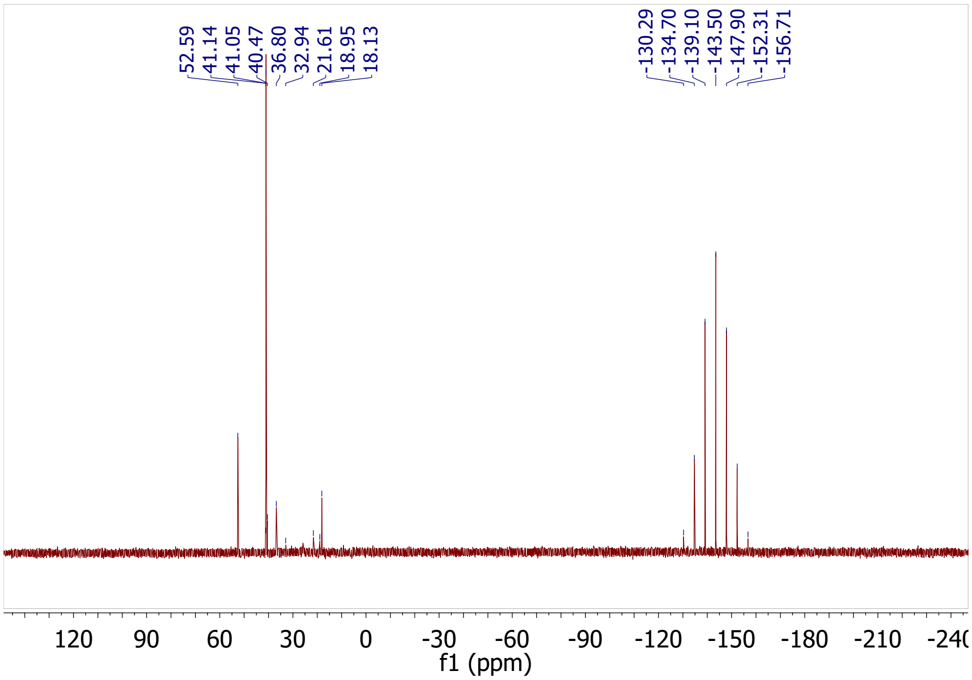

Figure S4: ^{31}P NMR (162 MHz, $CDCl_3$) spectra of compound **1'**.

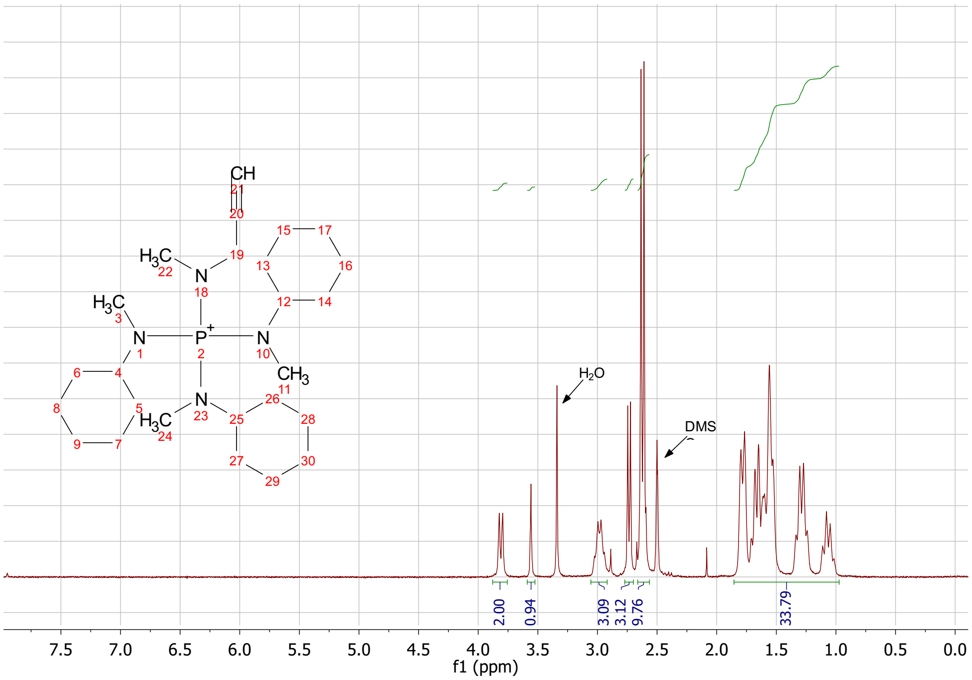

Figure S5: ^{1}H NMR (400 MHz, DMSO-d_6) spectra of compound **1**.

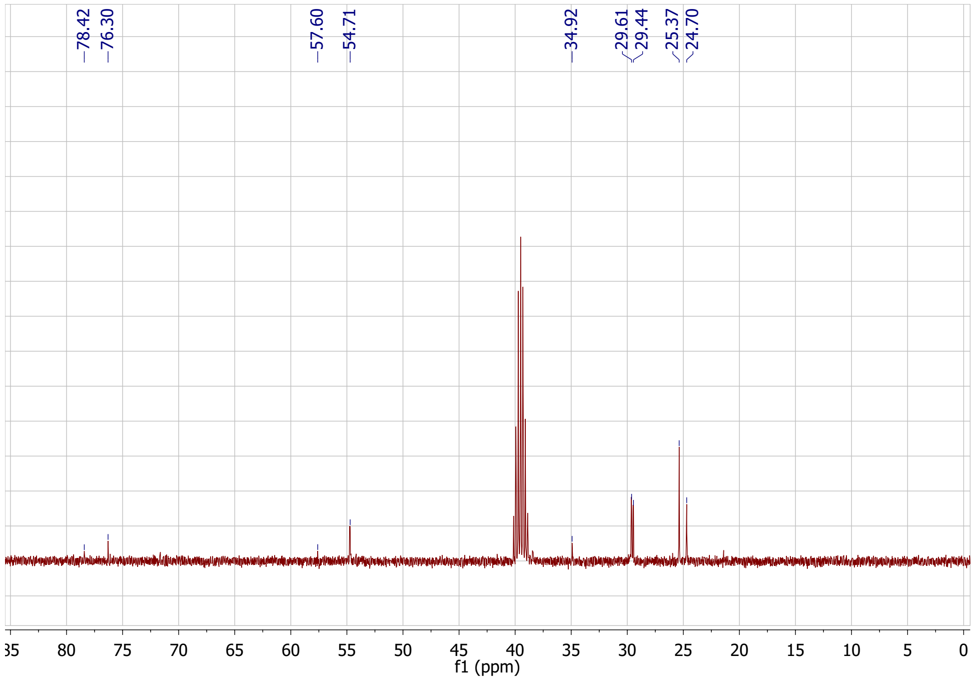

Figure S6: ^{13}C NMR (101 MHz, DMSO-d_6) spectra of compound **1**.

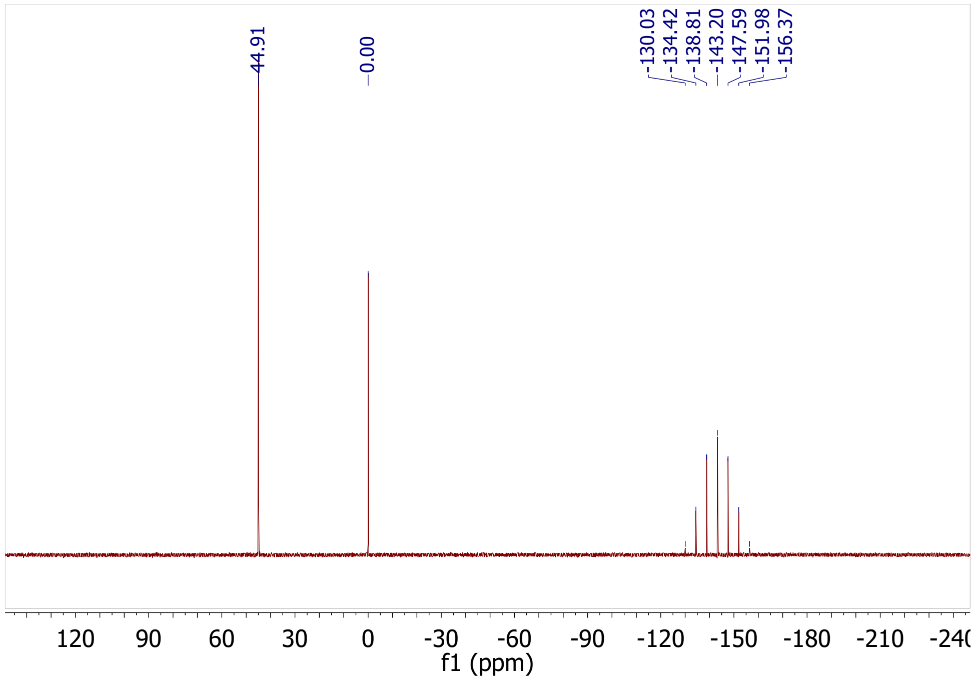

Figure S7: ^{31}P NMR (162 MHz, DMSO-d_6) spectra of compound **1**.

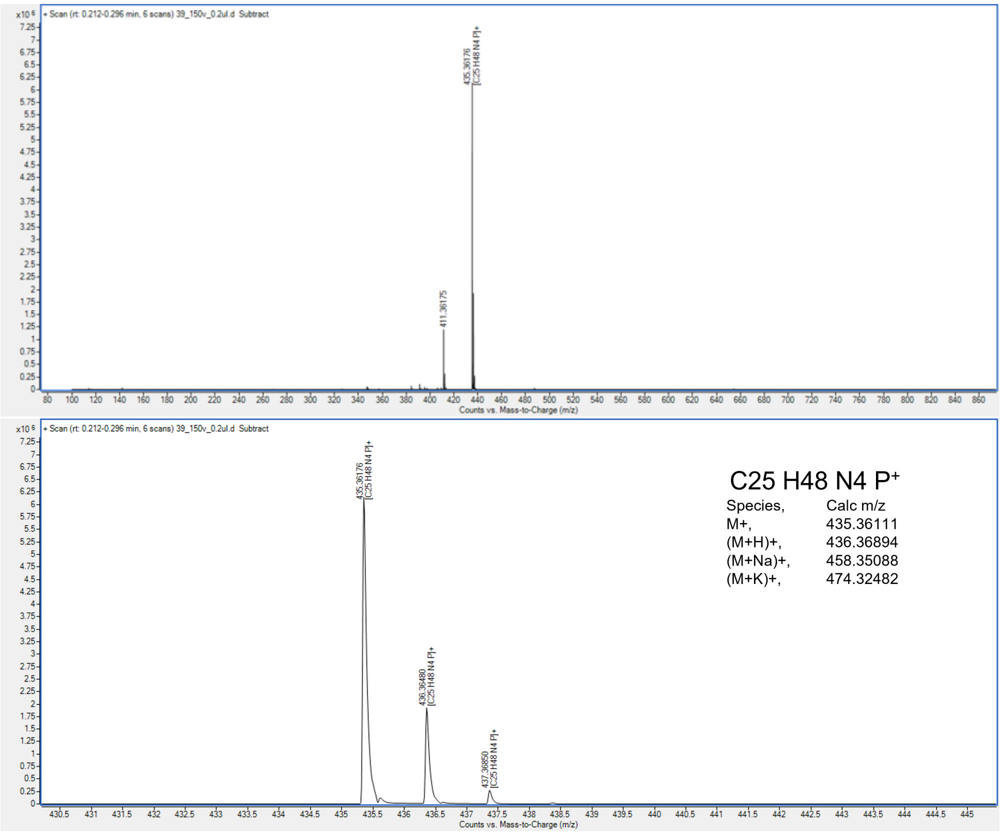

Figure S8: MS spectra of compound **1**.

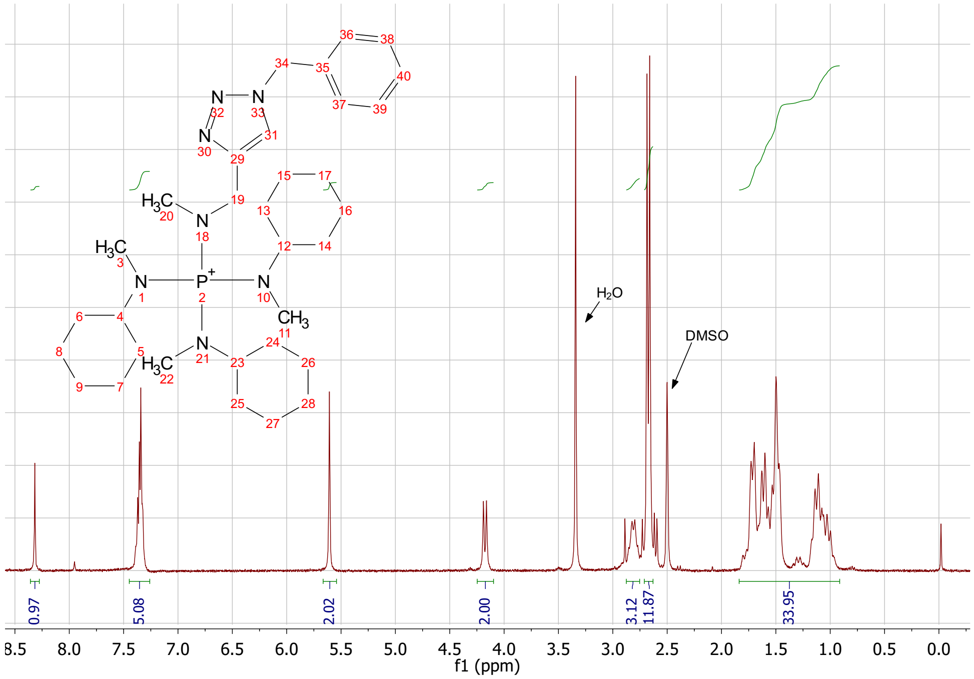

Figure S9: ^{1}H NMR (400 MHz, DMSO-d_6) spectra of compound **1a**.

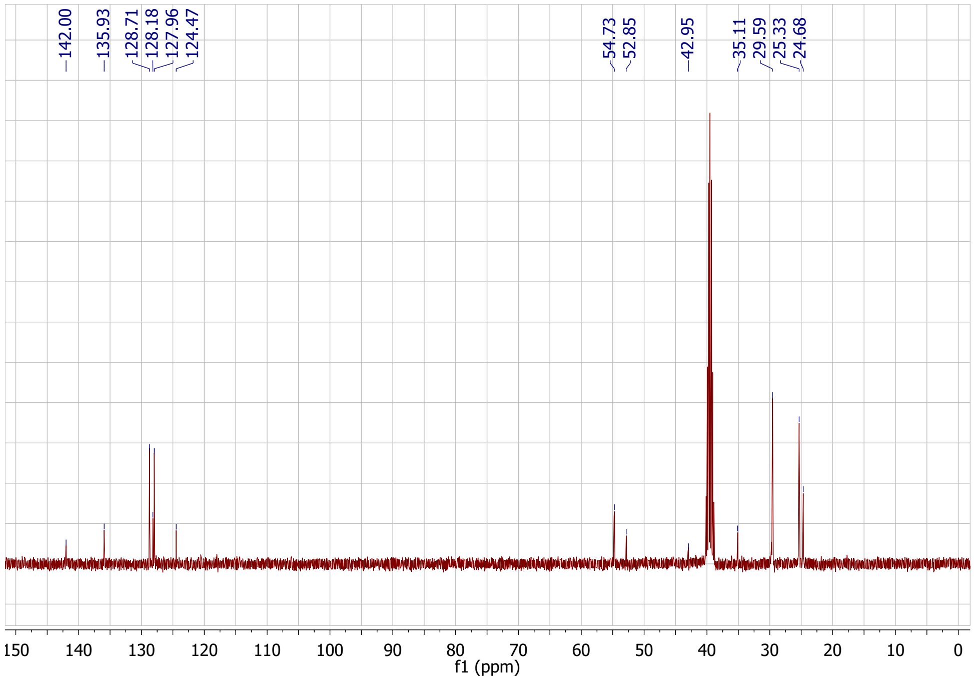

Figure S10: ^{13}C NMR (101 MHz, DMSO-d_6) spectra of compound **1a**.

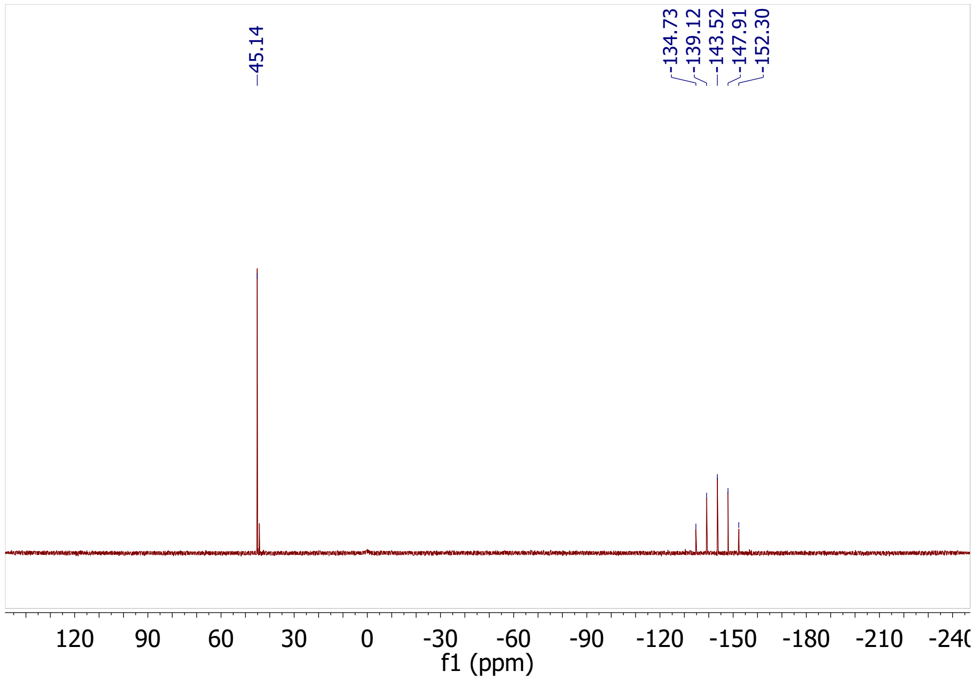

Figure S11: ^{31}P NMR (162 MHz, DMSO-d_6) spectra of compound **1a**.

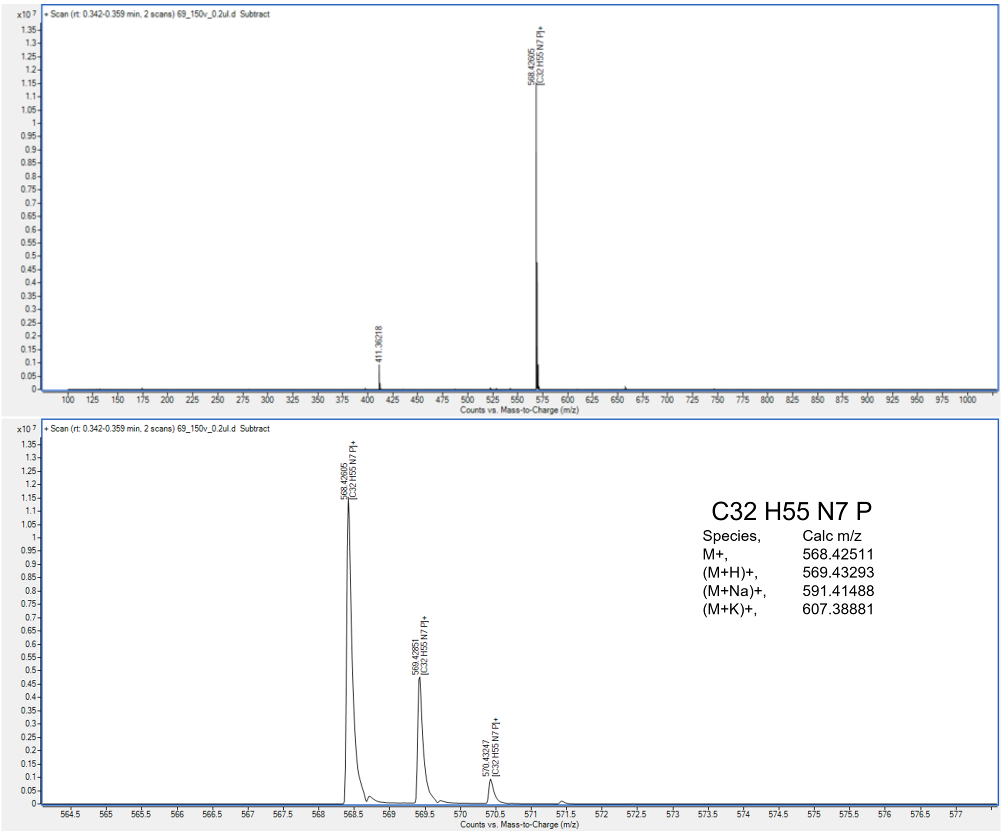

Figure S12: MS spectra of compound **1a**.

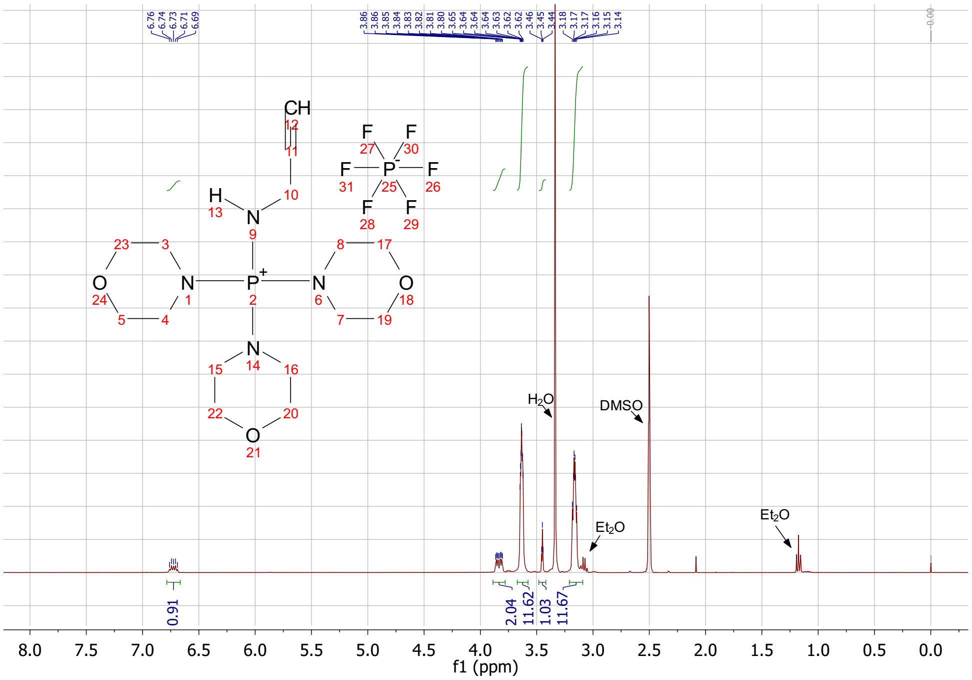

Figure S13: ^{1}H NMR (400 MHz, DMSO-d_6) spectra of compound **2'**.

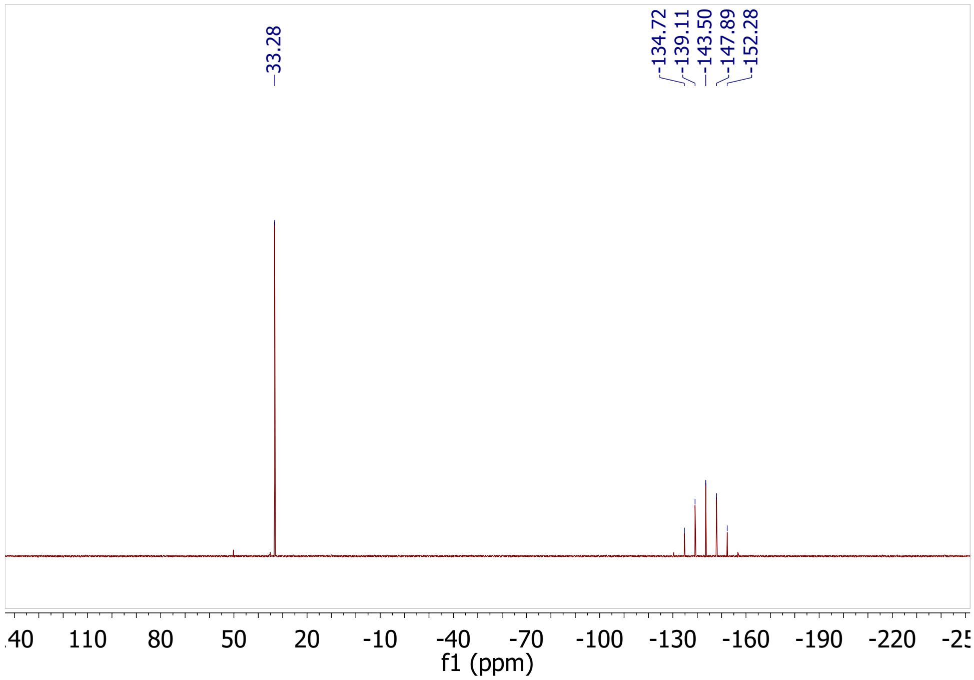

Figure S14: ^{31}P NMR (162 MHz, DMSO-d_6) spectra of compound **2'**.

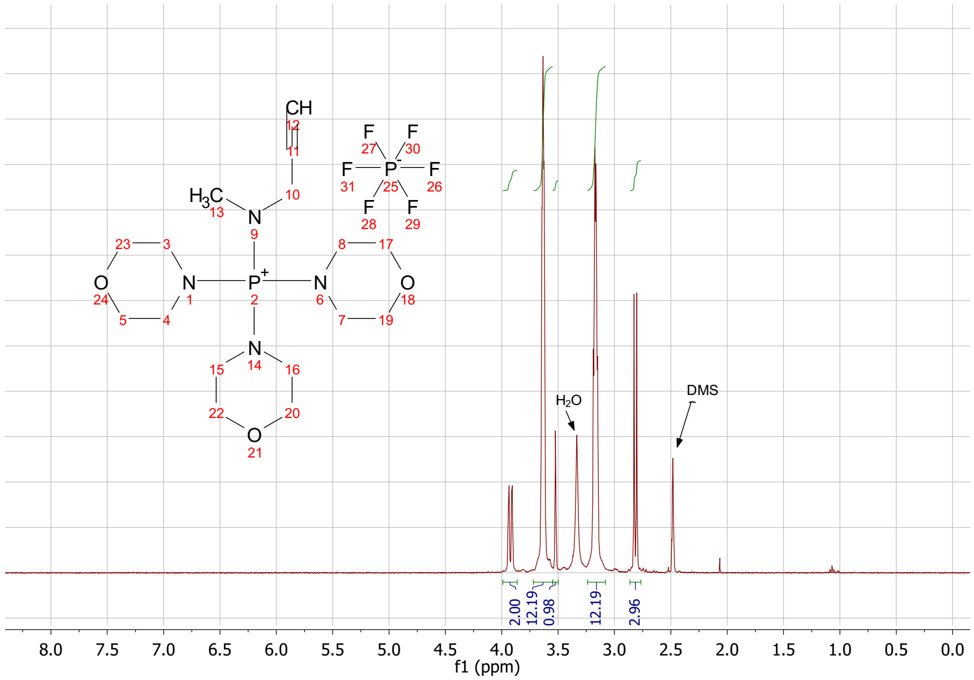

Figure S15: ^{1}H NMR (400 MHz, DMSO-d_6) spectra of compound **2**.

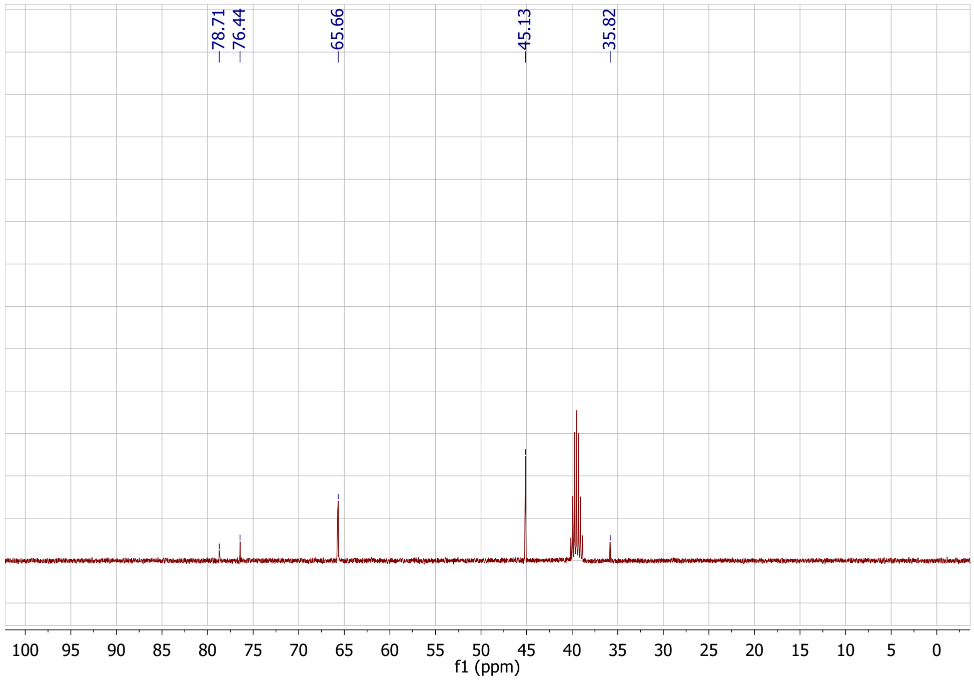

Figure S16: ^{13}C NMR (101 MHz, DMSO-d_6) spectra of compound **2**.

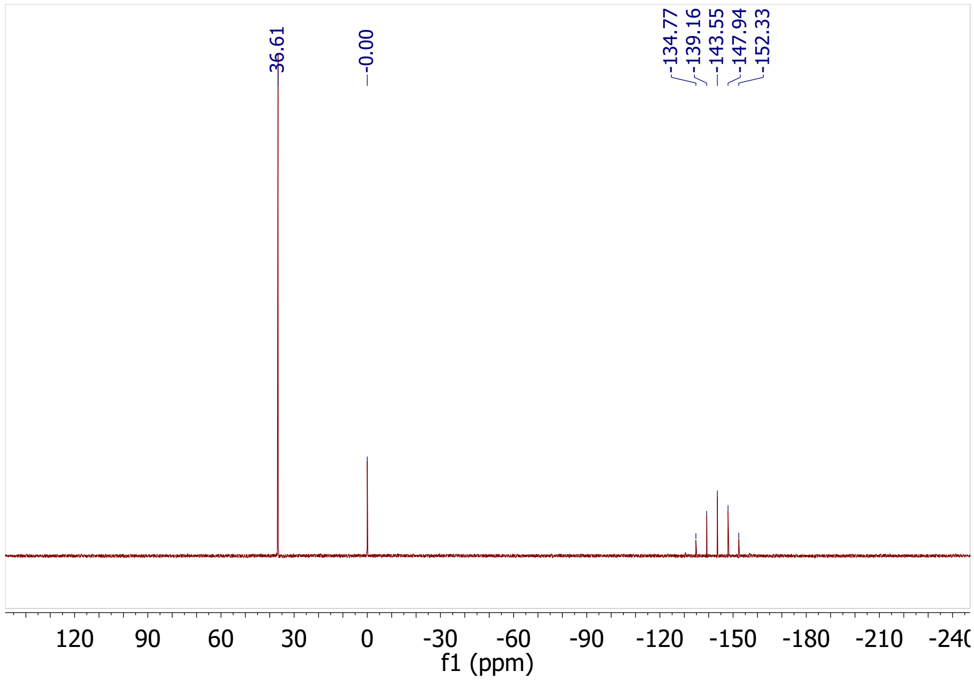

Figure S17: ^{31}P NMR (162 MHz, DMSO-d_6) spectra of compound **2**.

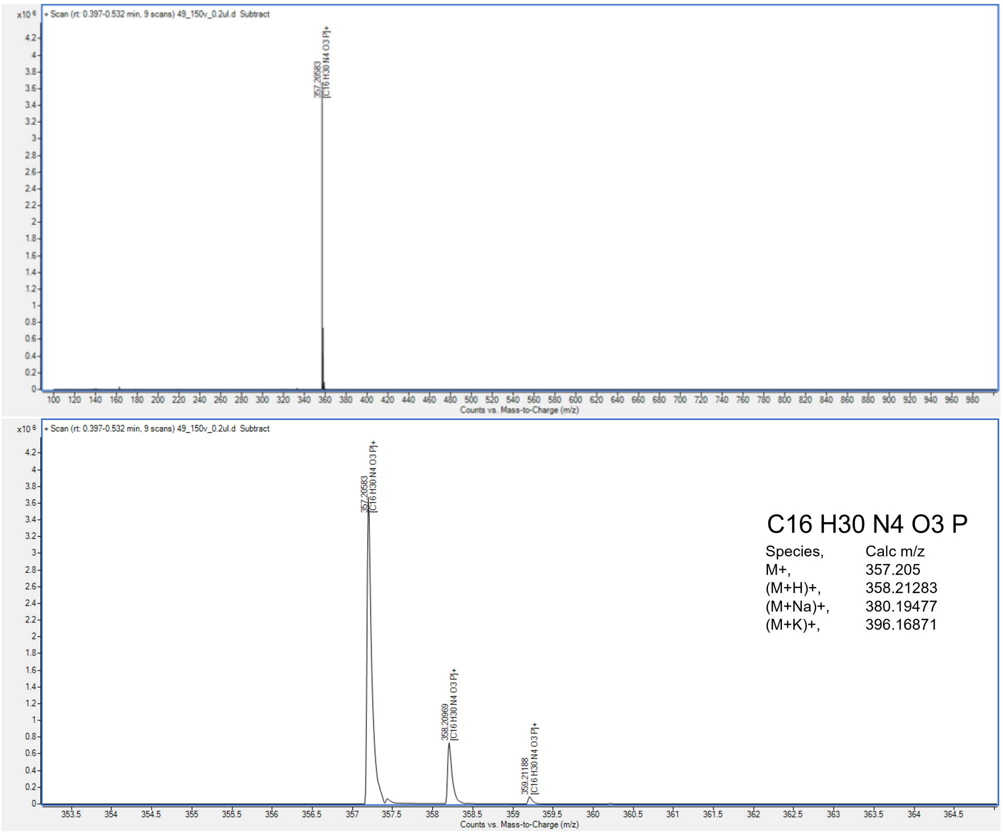

Figure S18: MS spectra of compound **2**.

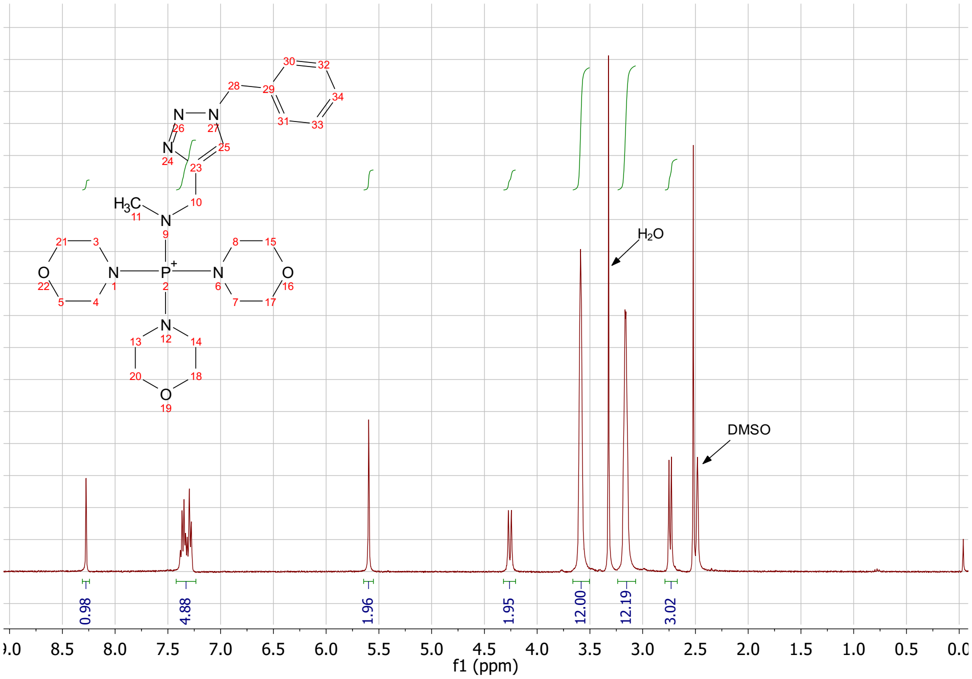

Figure S19: ^{1}H NMR (400 MHz, DMSO-d_6) spectra of compound **2a**.

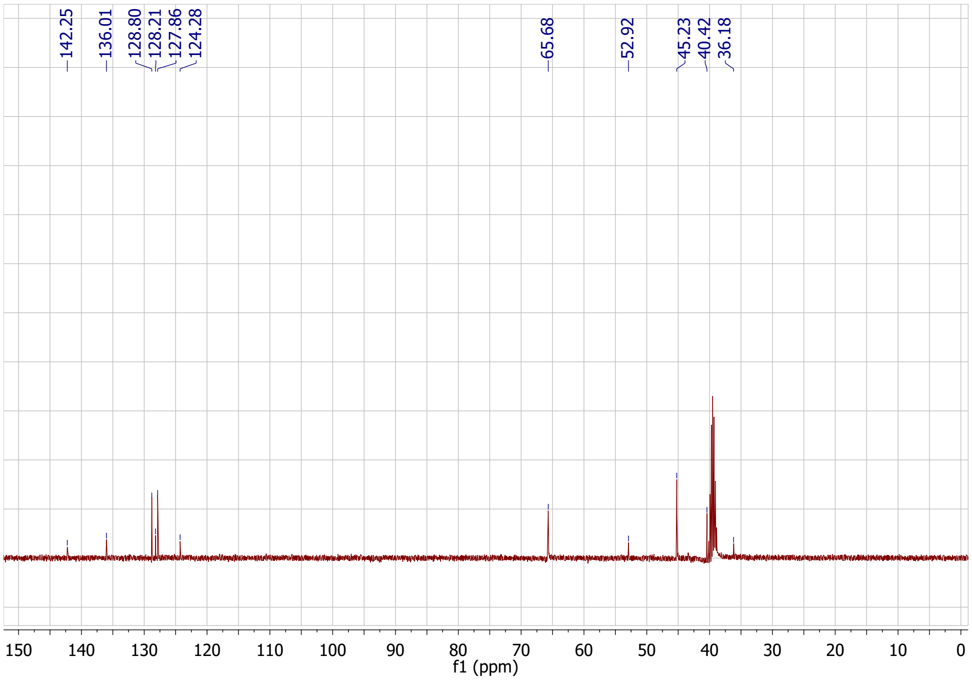

Figure S20: ^{13}C NMR (101 MHz, DMSO-d_6) spectra of compound **2a**.

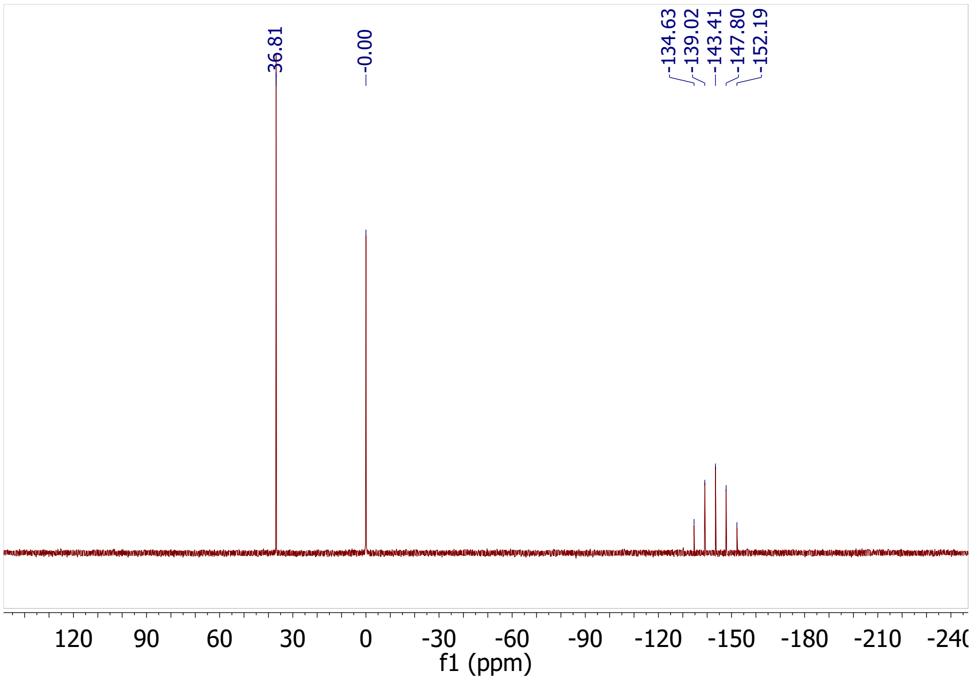

Figure S21: ^{31}P NMR (162 MHz, DMSO-d_6) spectra of compound **2a**.

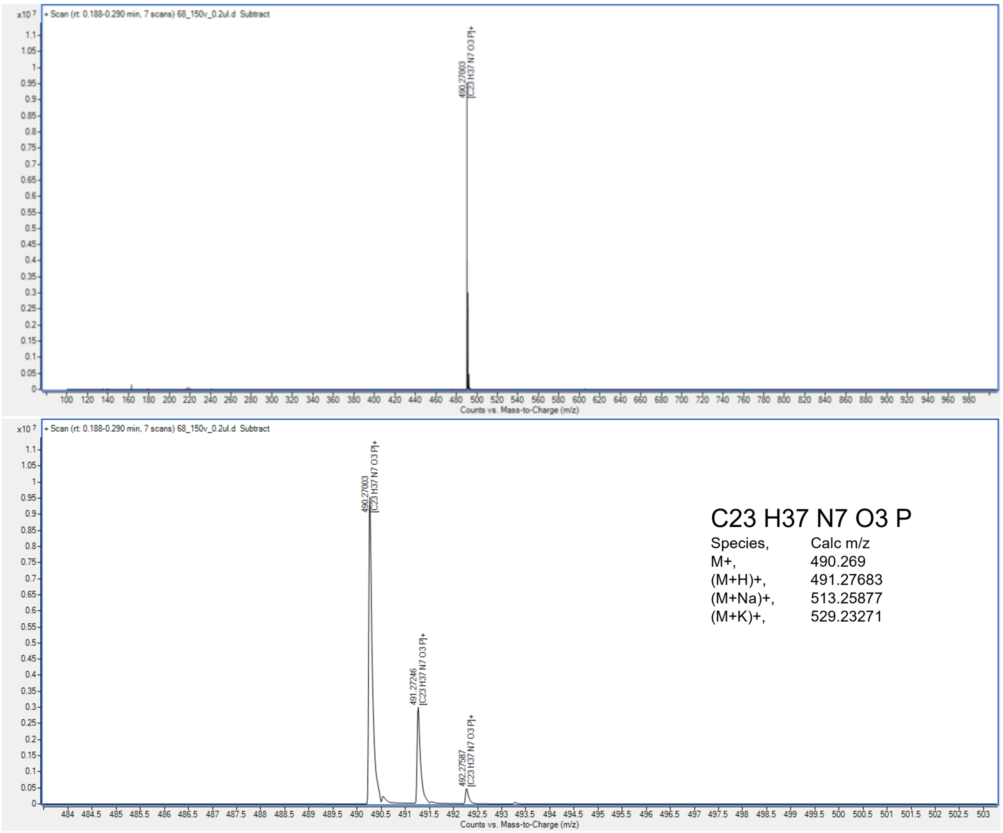

Figure S22: MS spectra of compound **2a**.

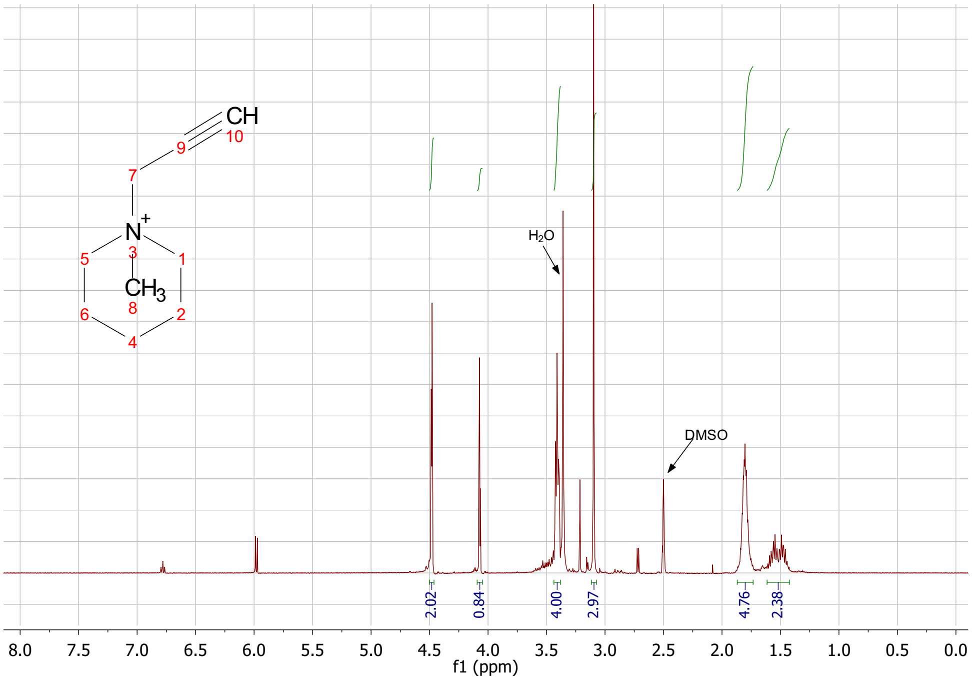

Figure S23: ^{1}H NMR (400 MHz, DMSO-d_6) spectra of compound **3**.

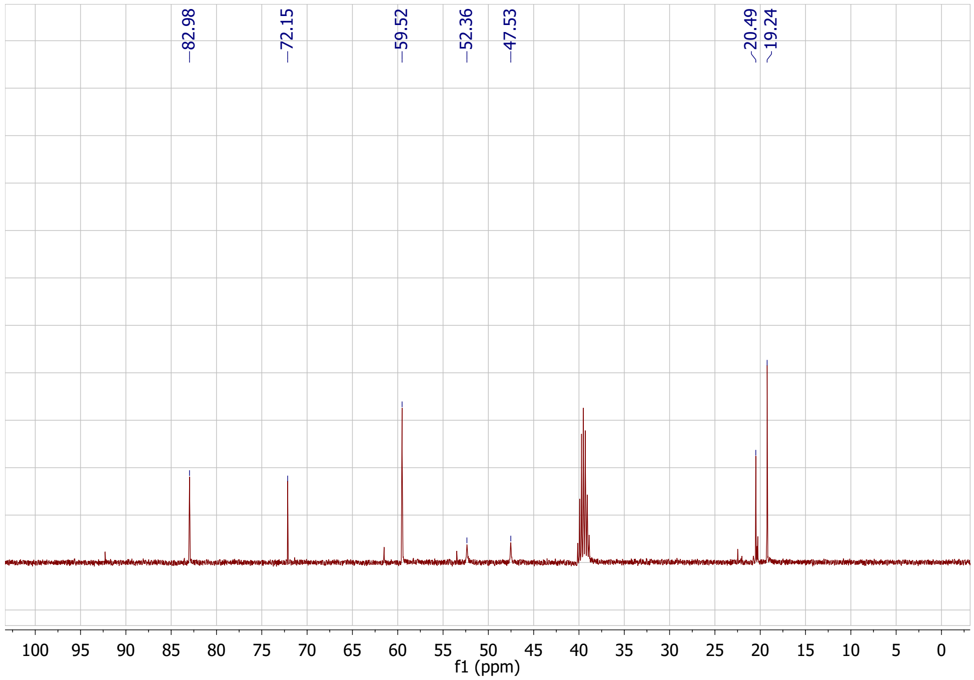

Figure S24: ^{13}C NMR (101 MHz, DMSO-d_6) spectra of compound **3**.

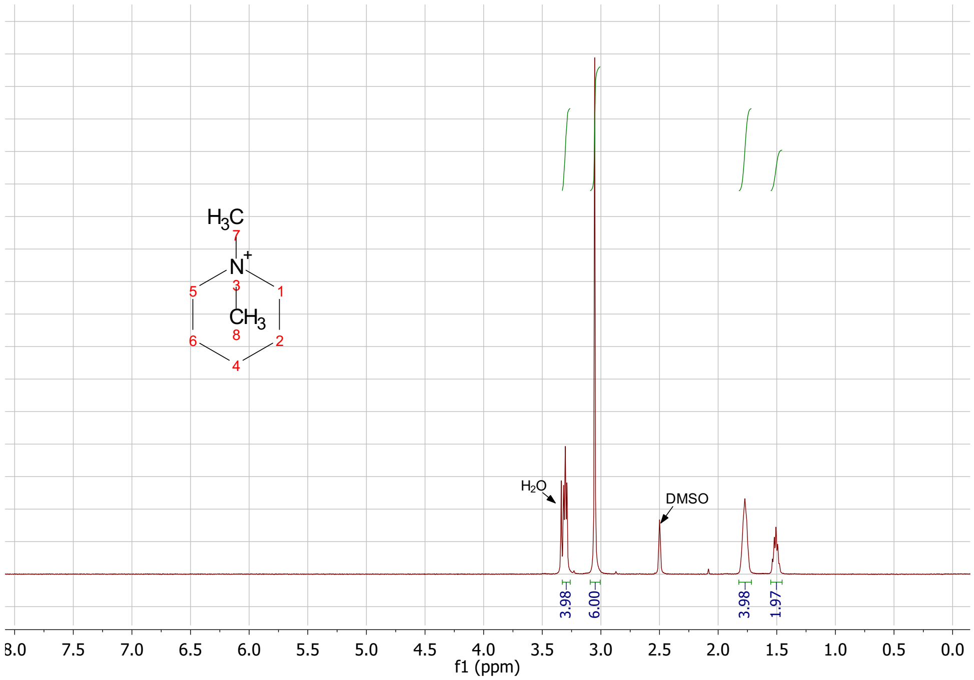

Figure S25: ^{1}H NMR (400 MHz, DMSO-d_6) spectra of compound **3a**.

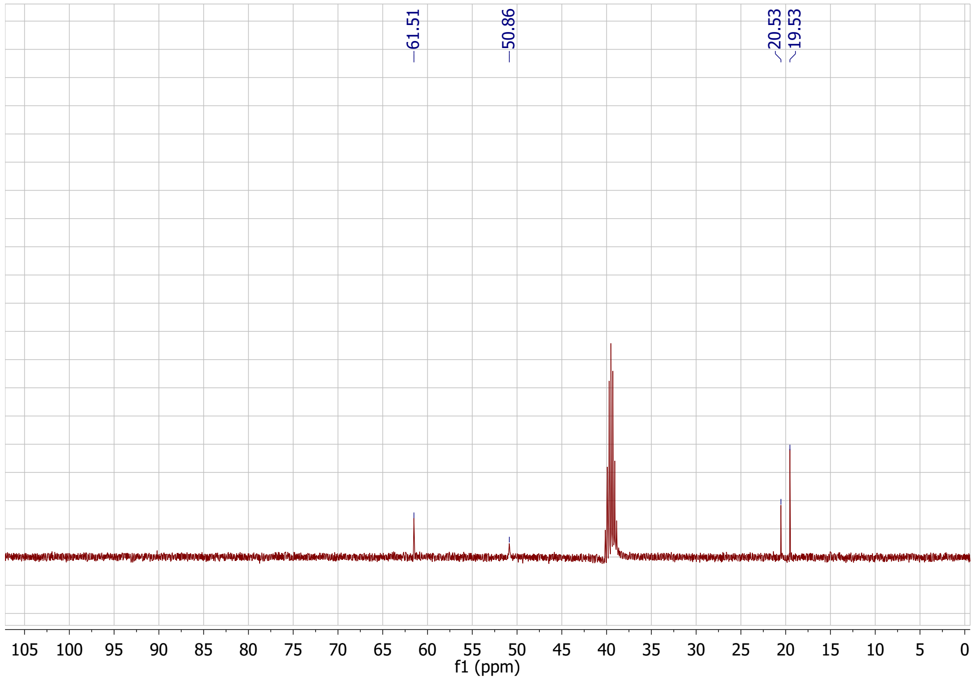

Figure S26: ^{13}C NMR (101 MHz, DMSO-d_6) spectra of compound **3a**.

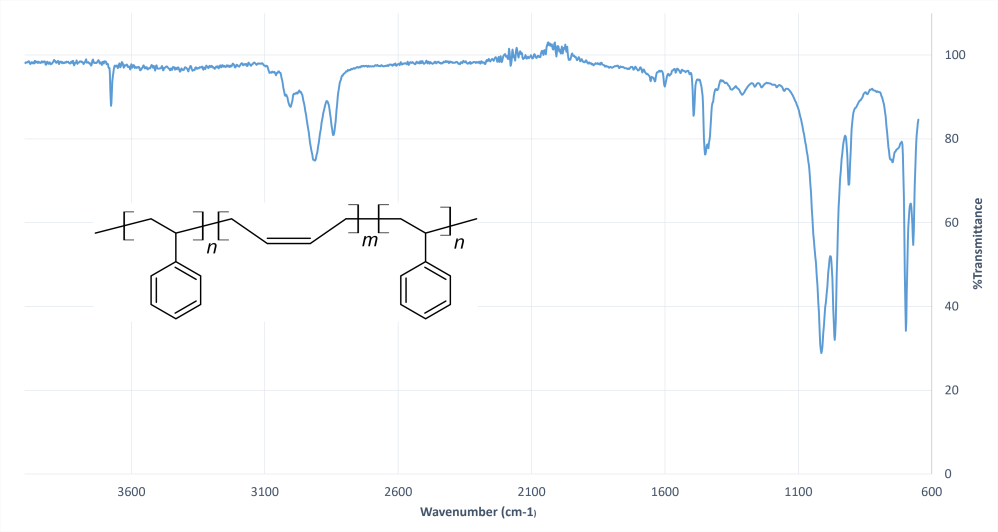

Figure S27: FTIR spectra of SBS.

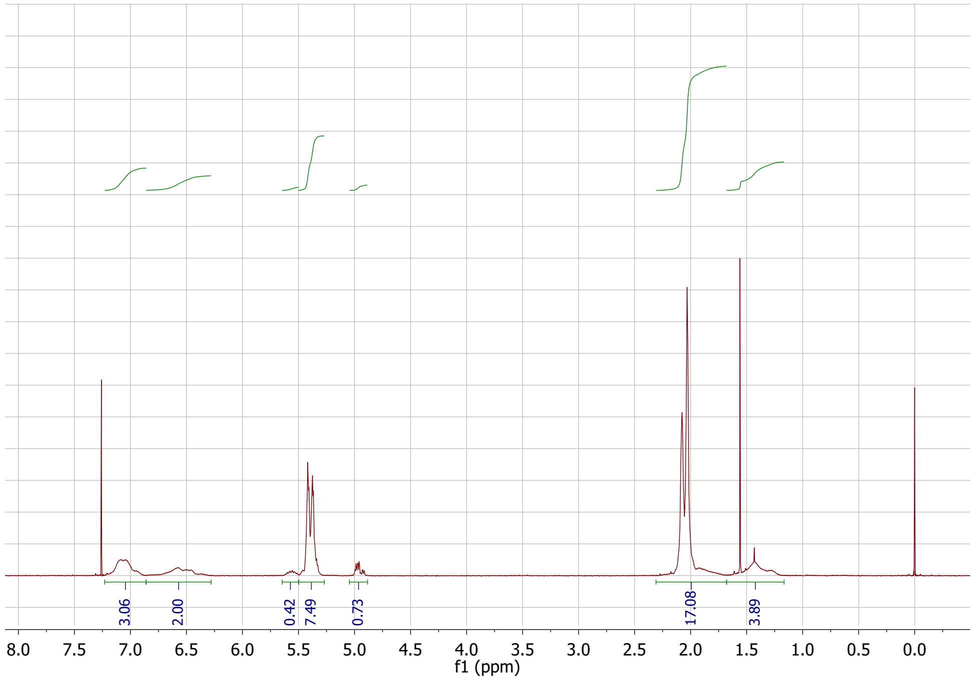

Figure S28: ^{1}H NMR (400 MHz, $CDCl_3$) spectra of SBS.

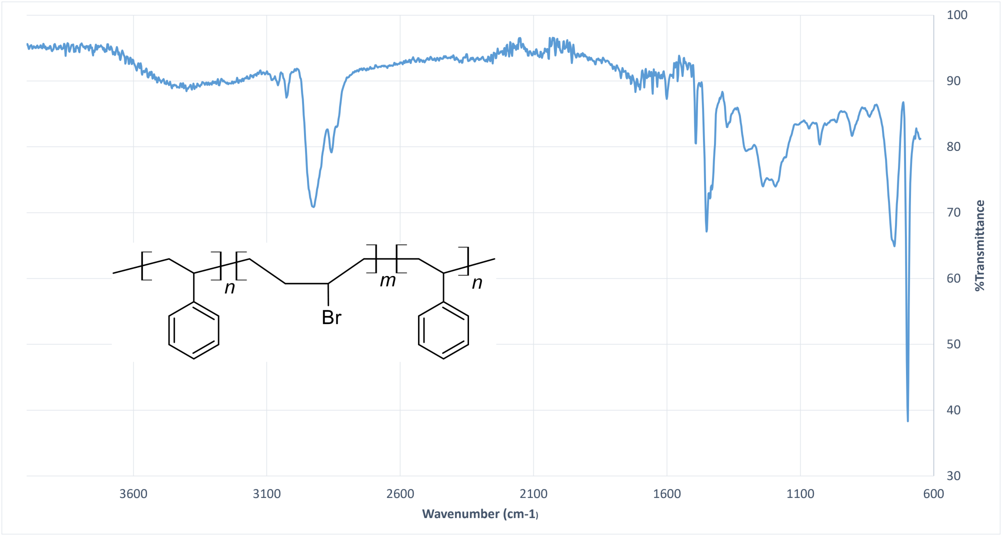

Figure S29: FTIR spectra of SBS-Br.

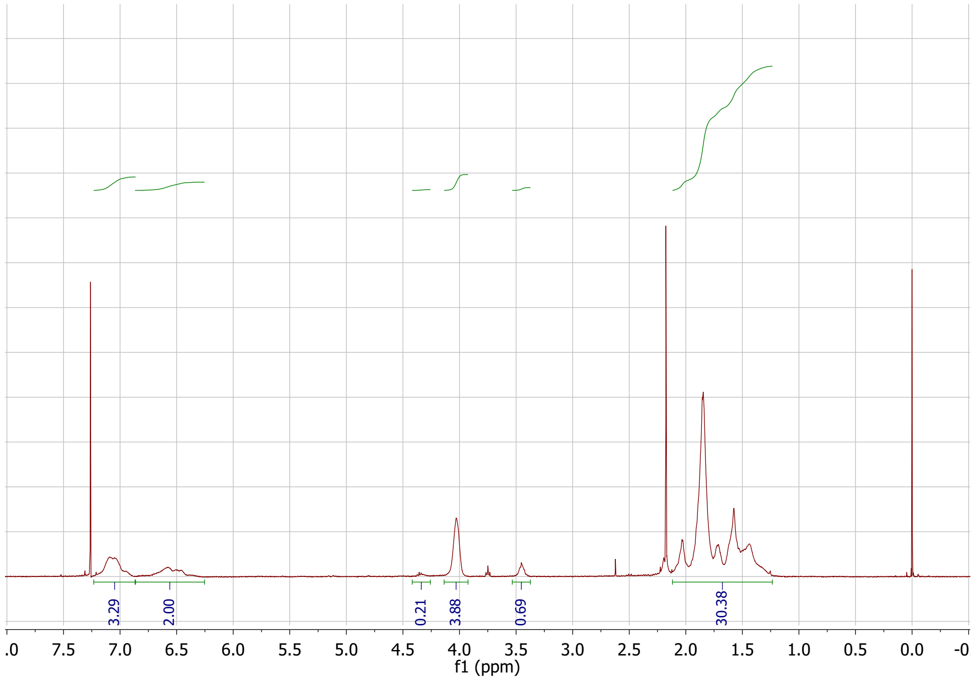

Figure S30: ^{1}H NMR (400 MHz, $CDCl_3$) spectra of SBS-Br.

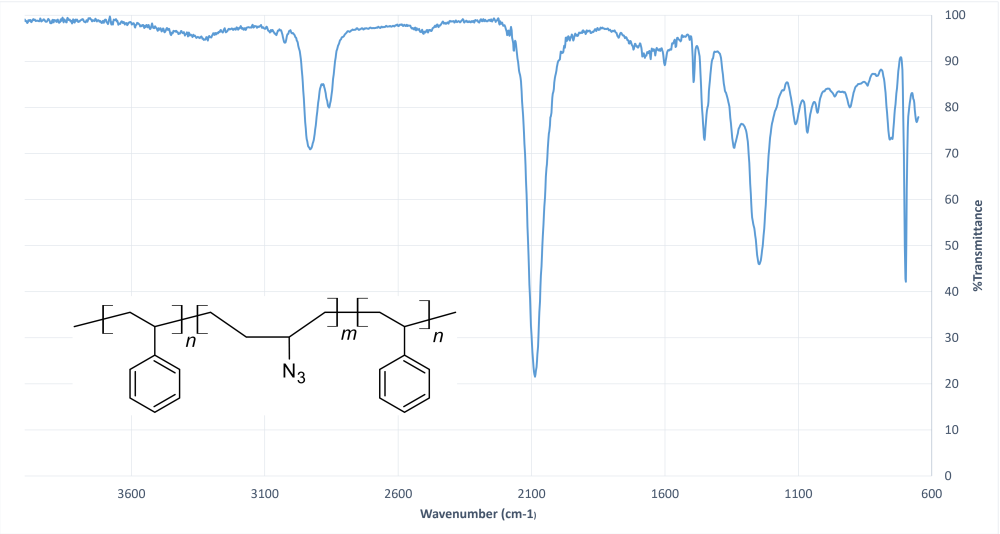

Figure S31: FTIR spectra of SBS-N3.

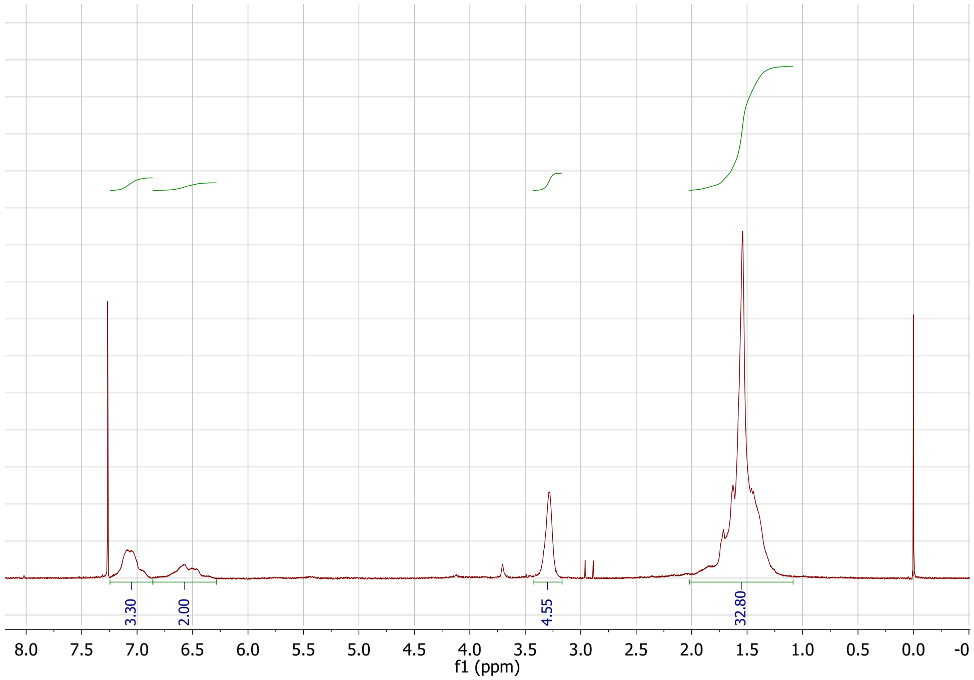

Figure S32: ^{1}H NMR (400 MHz, $CDCl_3$) spectra of SBS-N3.

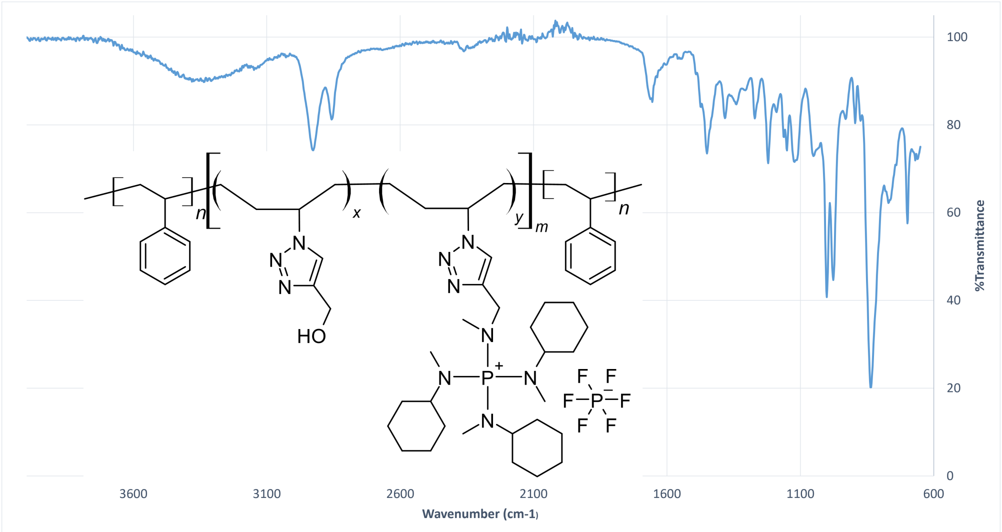

Figure S33: FTIR spectra of polyelectrolyte SBS-c-**1**A.

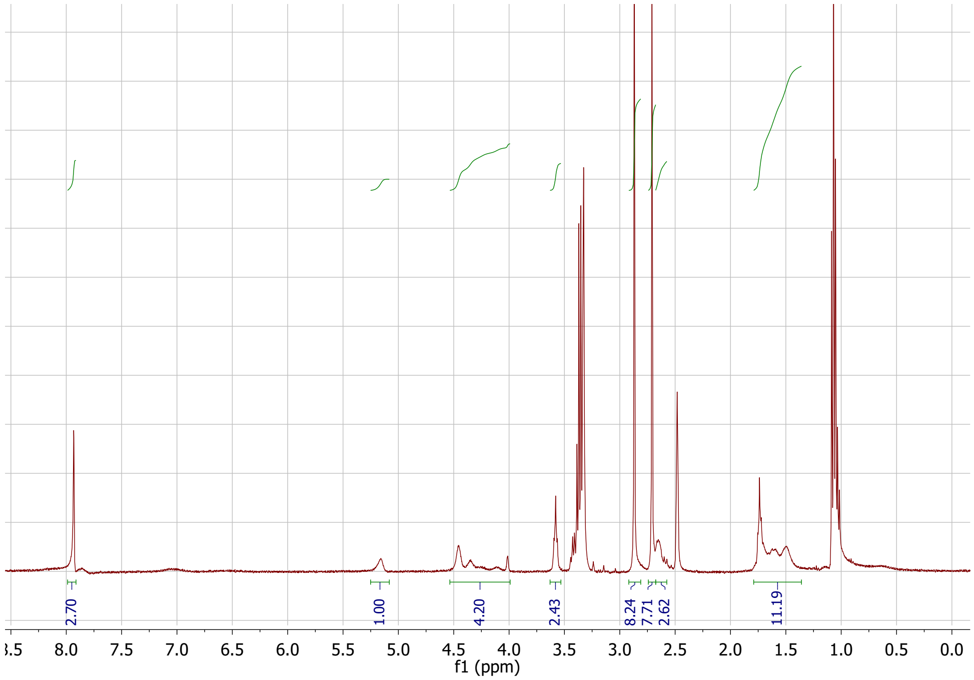

Figure S34: ^{1}H NMR (400 MHz, DMSO-d_6) spectra of polyelectrolyte SBS-c-**1**A.

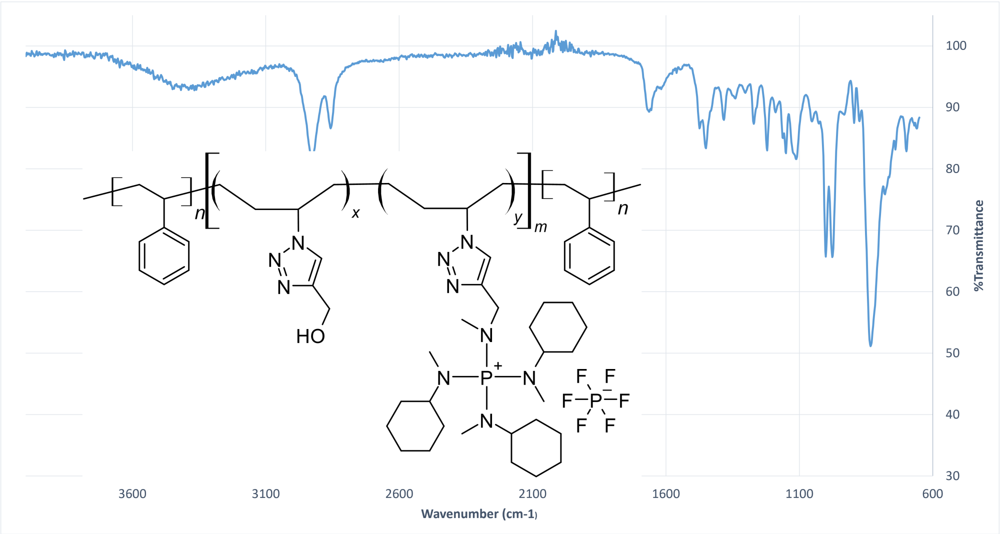

Figure S35: FTIR spectra of polyelectrolyte SBS-c-**1**B.

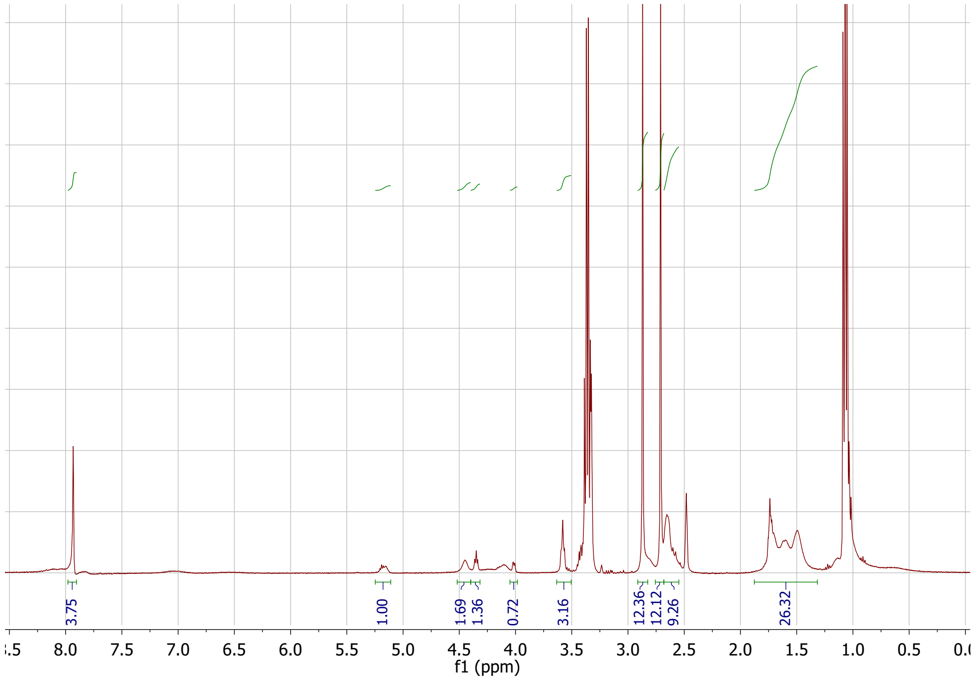

Figure S36: ^{1}H NMR (400 MHz, DMSO-d_6) spectra of polyelectrolyte SBS-c-**1**B.

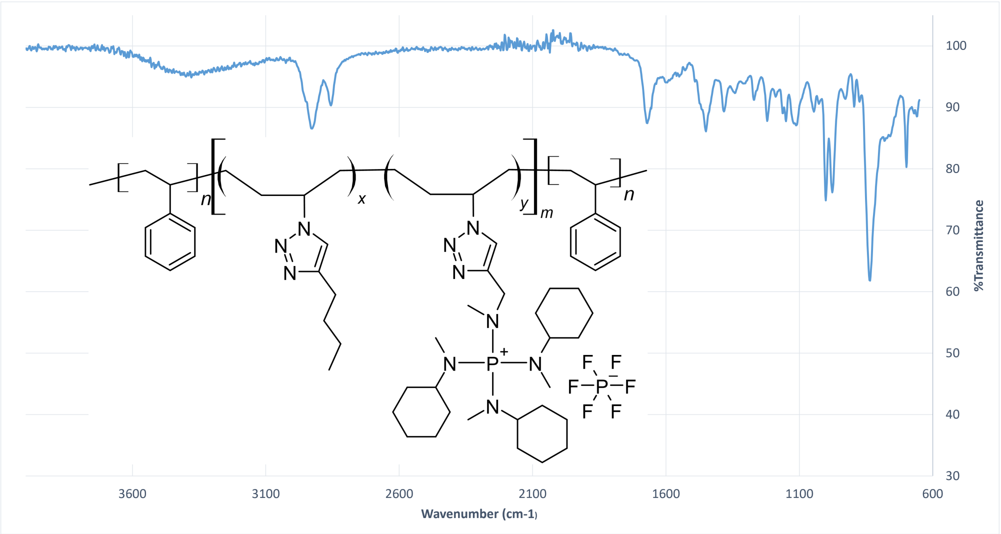

Figure S37: FTIR spectra of polyelectrolyte SBS-c-**1**C.

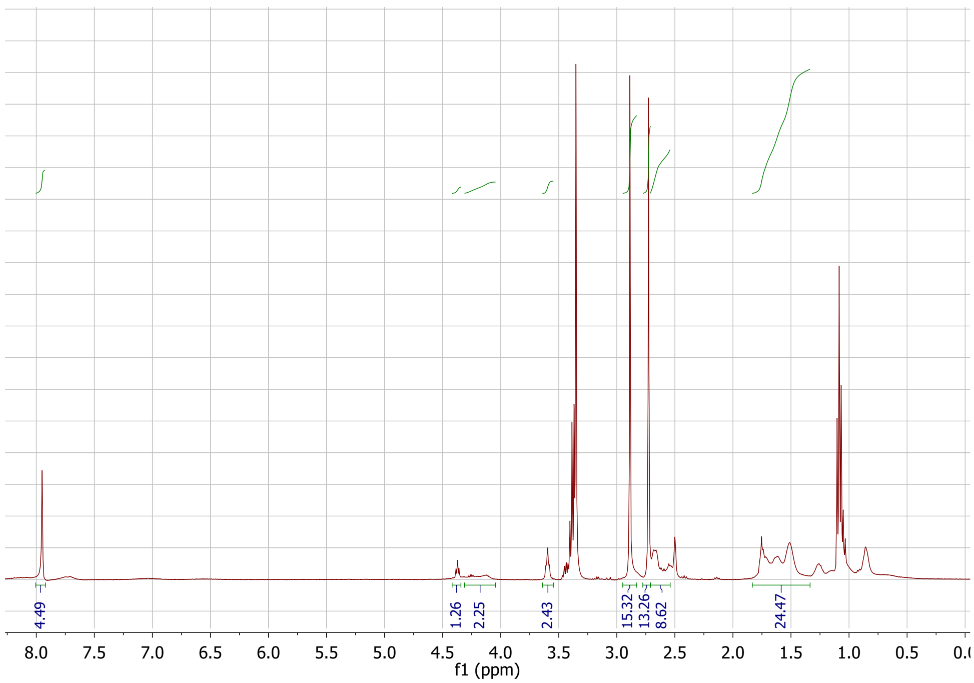

Figure S38: ^{1}H NMR (400 MHz, DMSO-d_6) spectra of polyelectrolyte SBS-c-**1**C.

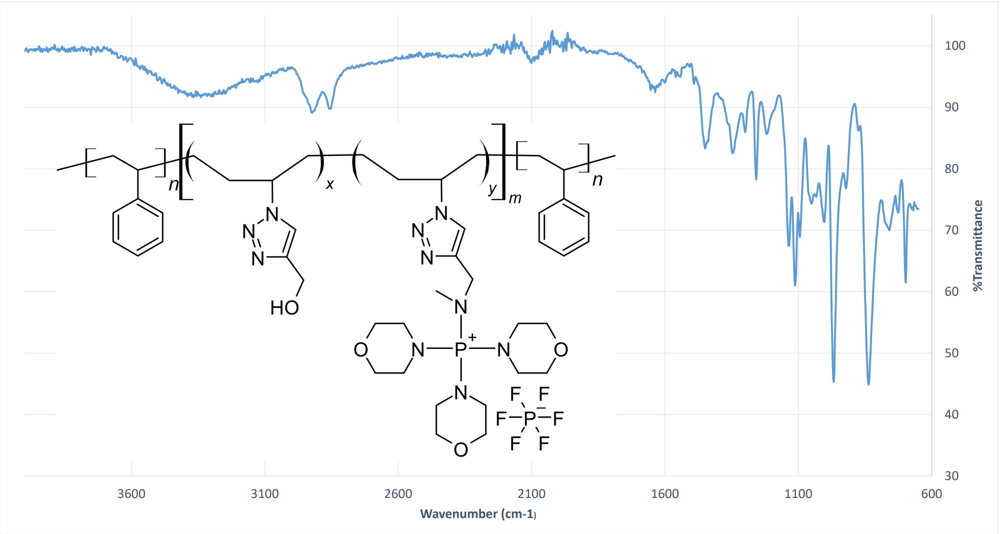

Figure S39: FTIR spectra of polyelectrolyte SBS-c-**2**A.

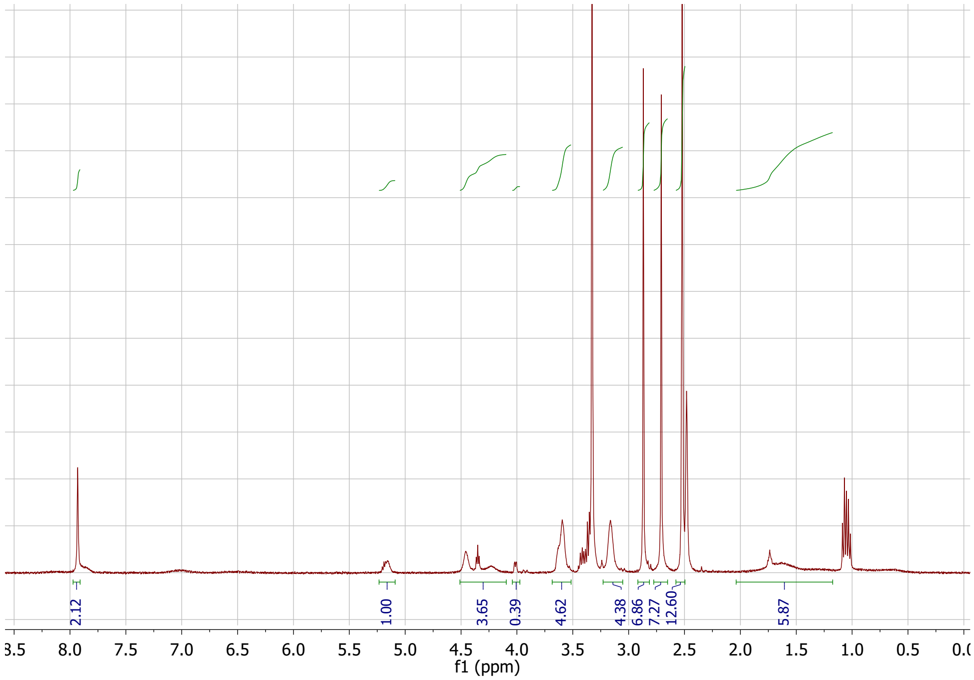

Figure S40: ^{1}H NMR (400 MHz, DMSO-d_6) spectra of polyelectrolyte SBS-c-**2**A.

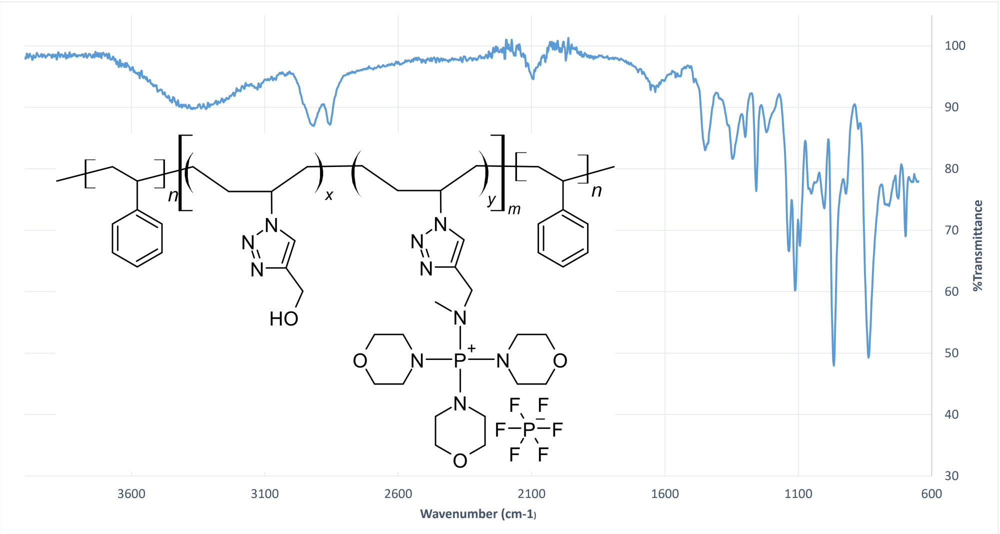

Figure S41: FTIR spectra of polyelectrolyte SBS-c-**2**B.

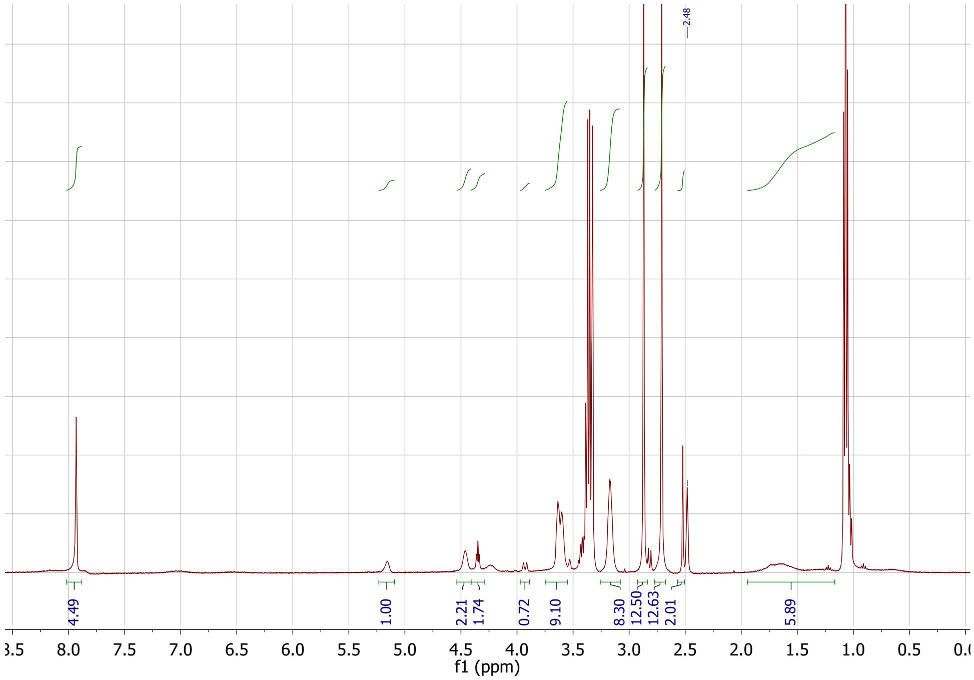

Figure S42: ^{1}H NMR (400 MHz, DMSO-d_6) spectra of polyelectrolyte SBS-c-**2**B.

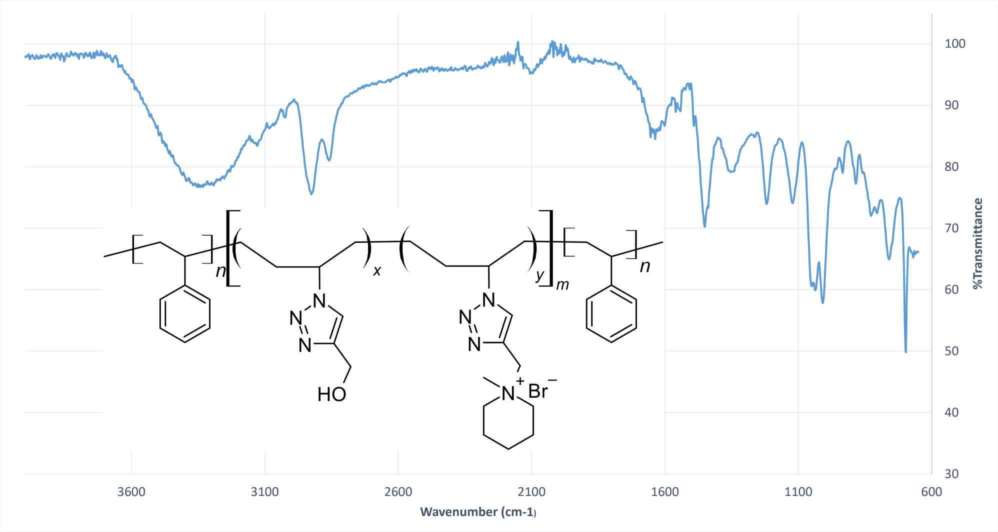

Figure S43: FTIR spectra of polyelectrolyte SBS-c-**3**A.

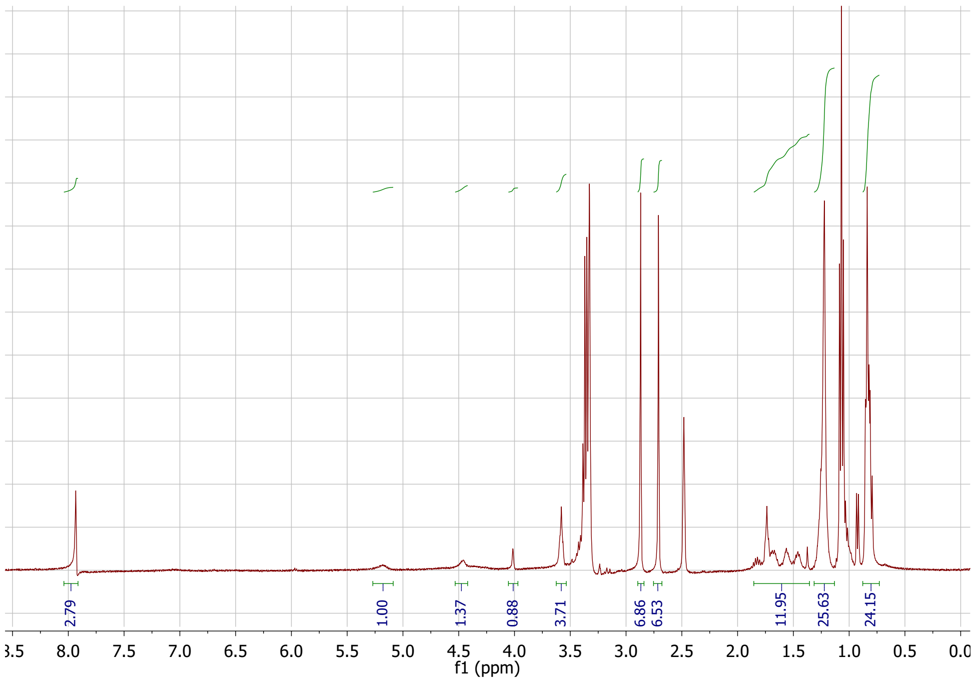

Figure S44: ^{1}H NMR (400 MHz, DMSO-d_6) spectra of polyelectrolyte SBS-c-**3**A.

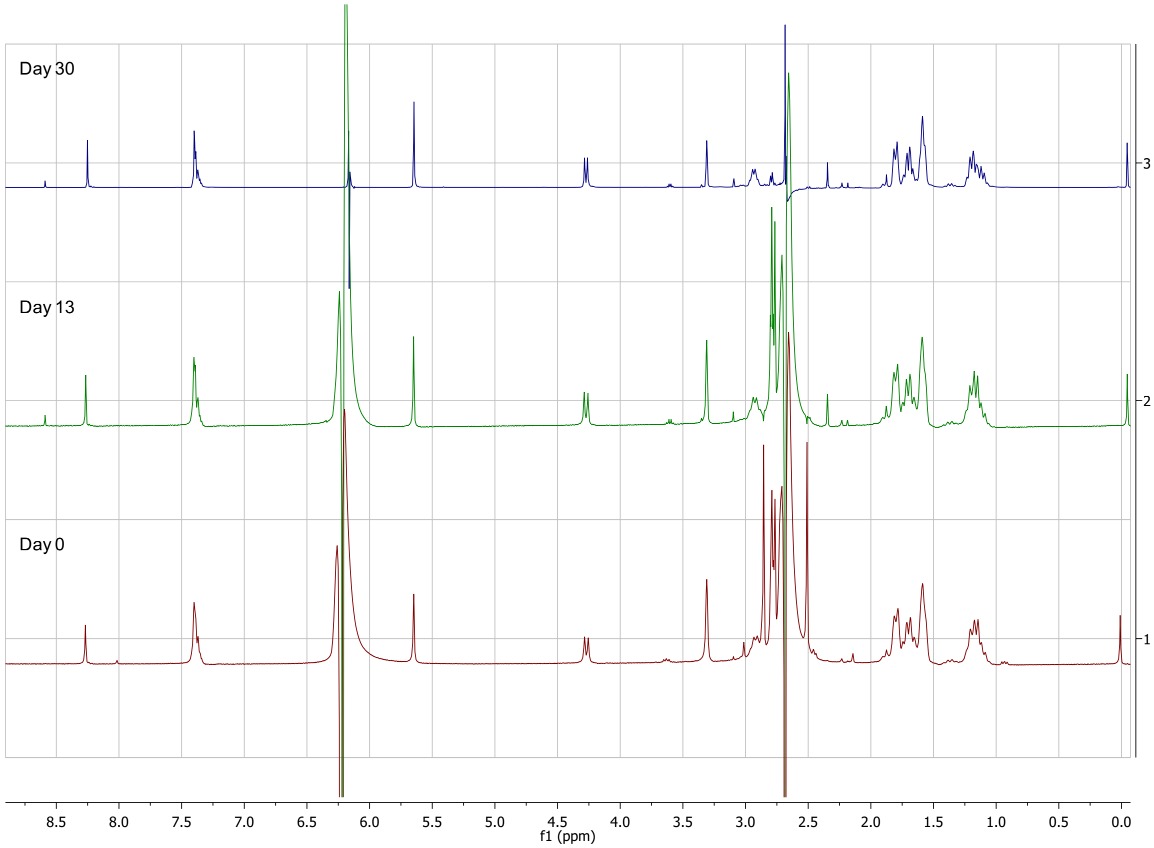

Figure S45: ^{1}H NMR (400 MHz, CD3OH) spectra of compound **1a** in 2 M KOH over 30 days. There was no observable change indicating the compound was very stable under alkaline conditions.

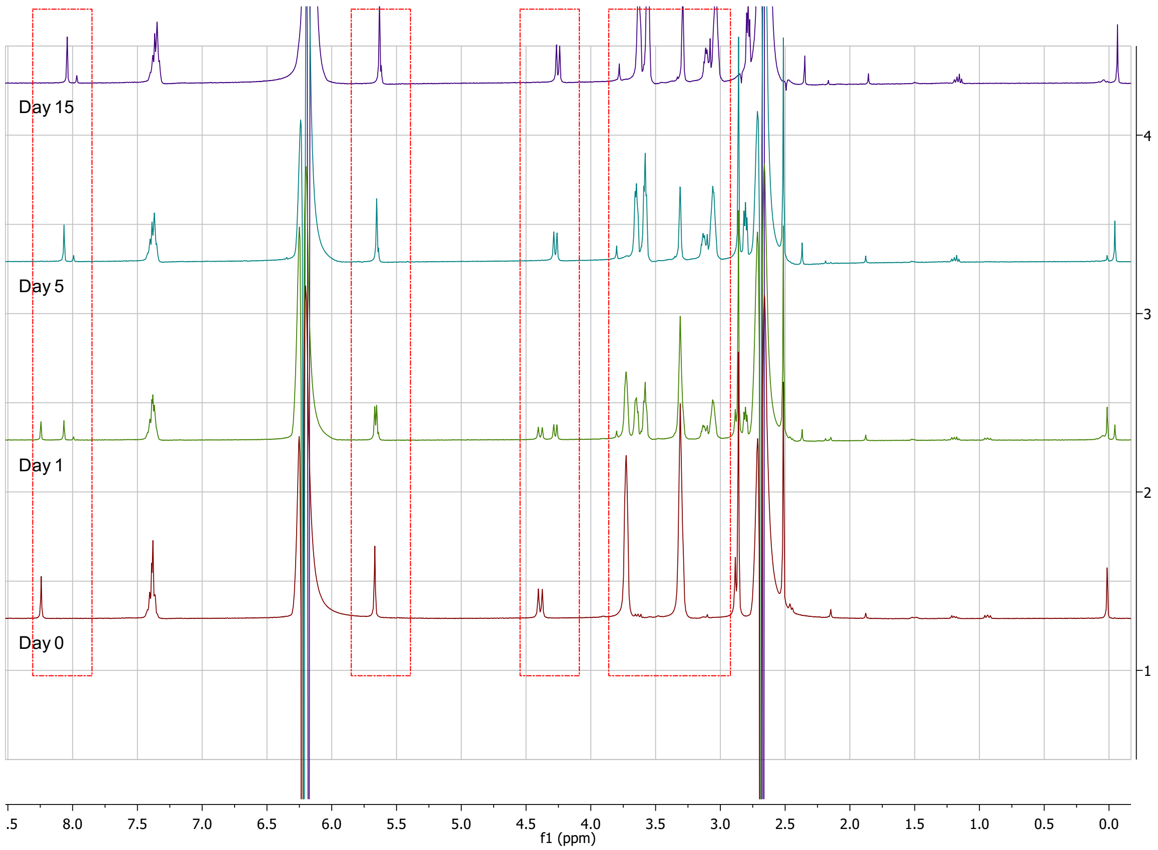

Figure S46: ^{1}H NMR (400 MHz, CD3OH) spectra of compound **2a** in 2 M KOH over 15 days. Half of the compound degraded within 1 day. Nearly all of the compound degraded in 5 days indicating the compound was very unstable under alkaline conditions.

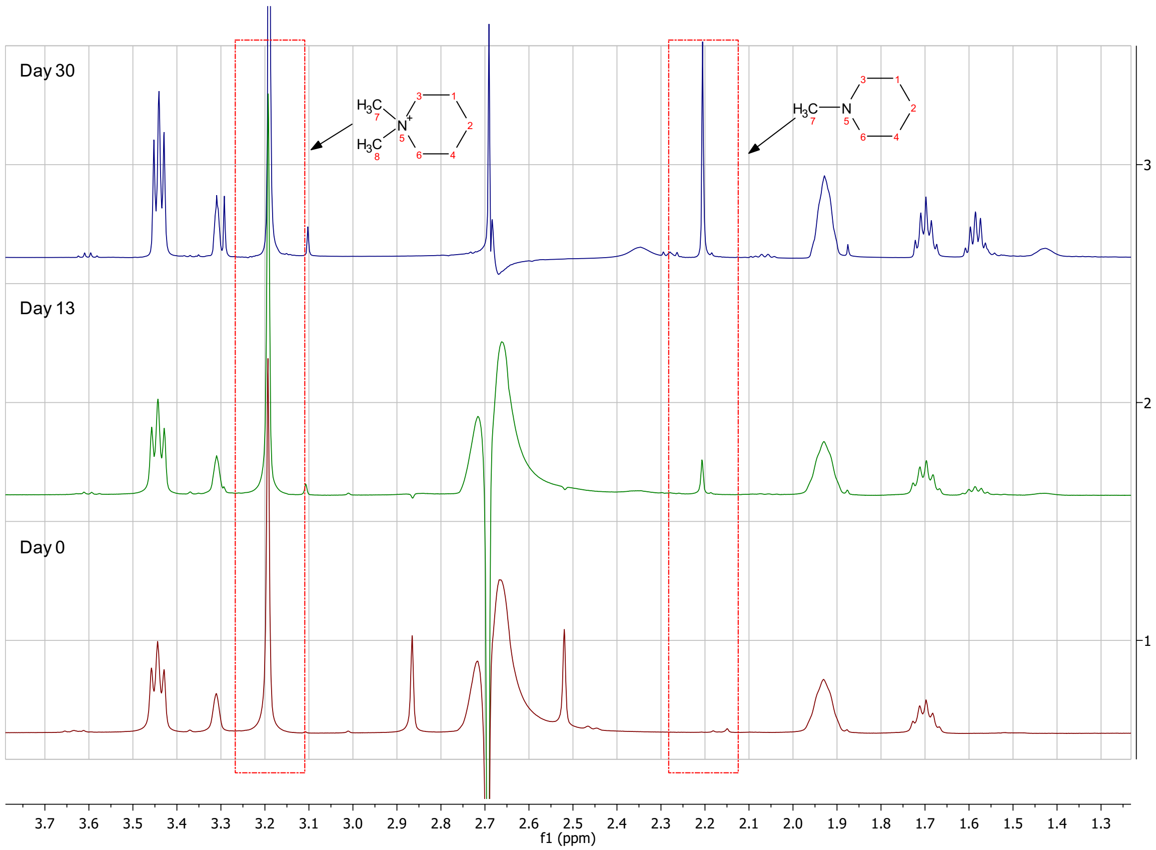

Figure S47: ^{1}H NMR (400 MHz, CD3OH) spectra of compound **3a** in 2 M KOH over 30 days. About 32% degradation of the compound was observed mainly by demethylation.

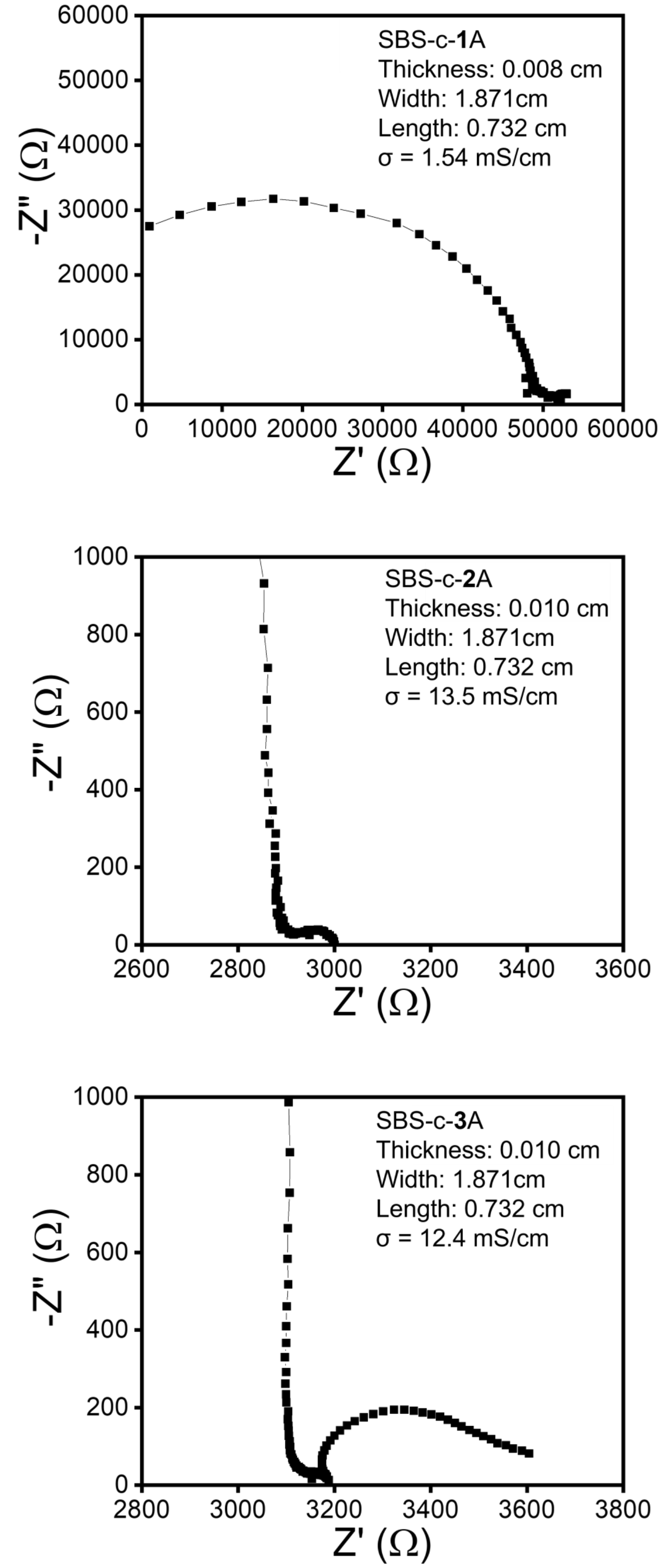

Figure S48: Ionic conductivity of the AEMs.
